# A systematic review investigating the role and impact of pharmacist interventions in cardiac rehabilitation

**DOI:** 10.1007/s11096-022-01517-1

**Published:** 2022-11-19

**Authors:** Aamna Ahmed, Ping Guo, Zahraa Jalal

**Affiliations:** 1 School of Pharmacy, Institute of Clinical Sciences, University of Birmingham, Birmingham, UK; 2 School of Nursing and Midwifery, Institute of Clinical Sciences, College of Medical and Dental Sciences, University of Birmingham, Birmingham, UK

**Keywords:** Cardiovascular diseases, Cardiac rehabilitation, Pharmacists, Pharmacist-led interventions, Secondary prevention, Systematic review

## Abstract

**Background:**

Cardiovascular disease (CVD) is a predominant cause of mortality. Pharmacists play an important role in secondary prevention of CVD, however, their role in cardiac rehabilitation is under-reported and services are under-utilised.

**Aim:**

To explore the role of pharmacists in cardiac rehabilitation, the impact of their interventions on patient outcomes, and prospects of future role development.

**Method:**

Databases searched were PubMed, Embase, Medline, Cochrane Library, Cumulative Index to Nursing and Allied Health Literature (CINAHL), and PsycINFO from January 2006 to October 2021. Randomised and non-randomised controlled trials were selected if they assessed the role of pharmacists in cardiac rehabilitation. Cochrane risk of bias tool, Joanna Briggs Institute (JBI) Critical Appraisal Tool for Quasi-Experimental Studies and the National Heart, Lung and Blood Institute (NIH) quality assessment tool, were used to assess quality and a narrative synthesis was conducted.

**Results:**

The search yielded 786 studies, only five met the inclusion criteria. The pharmacist-led interventions included patient education, medication review and reconciliation, and medication adherence encouragement. Four out of the five studies showed that pharmacist-led interventions in cardiac rehabilitation significantly improved patient clinical and non-clinical outcomes. One study showed a statistically significant reduction in low density lipoprotein-cholesterol (LDL-C) levels to optimal target of < 70 mg/dL (80% vs 60%, *p* = 0.0084). Two studies reported better medication adherence, and two studies showed greater improvement in all domains of health-related quality of life observed in the intervention group.

**Conclusion:**

Pharmacist-led interventions in cardiac rehabilitation could lower CVD risk factors and hence recurrence. Although these findings support pharmacists’ involvement in cardiac rehabilitation, larger intervention studies are needed to evaluate the feasibility of pharmacist-led interventions and their impact on hospital admissions and mortality risk.

**Supplementary Information:**

The online version contains supplementary material available at 10.1007/s11096-022-01517-1.

## Impact statements

- The evidence presented in this review provides an important message to healthcare organisations and policy makers regarding the effectiveness of pharmacist interventions in cardiac rehabilitation.
- Results from this review recommend further integration of pharmacist roles in cardiac rehabilitation as a strategy to improve patient outcomes, and to ease the burden on healthcare services.
- There was a paucity of studies investigating the role of pharmacists in cardiac rehabilitation, and further large intervention studies are needed to derive definitive conclusions on the impact of the pharmacy role.

## Introduction

Cardiovascular disease (CVD) is a major cause of death and accounts for an annual mortality rate of approximately 17.9 million worldwide [1]. Despite the high mortality rate related to CVD, the number of patients discharged from hospital following a cardiac event is increasing. Therefore, patients benefit from treatment to reduce manifestations of cardiac disease; this is known as secondary prevention. Secondary prevention of CVD includes pharmacological therapy and cardiac rehabilitation (CR) [2]. CR is a multidisciplinary (involvement of cardiologists, nurses, dieticians, pharmacists, occupational therapists) and multifactorial programme for patients who have either had a cardiac event (e.g., myocardial infarction, heart failure, angina) or undergone cardiac procedures such as bypass surgery, heart valve replacement, heart transplant, or coronary angioplasty/stent [3]. It aims to restore patient’s quality of life by improving health outcomes and thus preventing recurrence; this is often achieved through promoting healthy behaviours, medical risk factor management, and psychosocial therapies [2, 4]. As a result, CR could facilitate a reduction in hospital re-admission and create a positive impact on a macroeconomic level [4].

The provision of CR infers great clinical benefits to patients who have experienced myocardial infarction. Dalal et al. showed a reduction in 30-day mortality risk from 13 to 8% when patients were educated on the importance of adherence to treatment and taught to modify lifestyle factors [5]. A reduction in the risk of further cardiac events can also lead to an improvement in Health-Related Quality of Life (HRQoL) [6, 7]. A systematic review and meta-analysis of 63 studies demonstrated that patients with heart failure and arrhythmias who attended exercise-based CR had a reduction in CVD mortality (RR 0.74; CI 0.64–0.86) and hospital admission (RR 0.82; CI 0.70–0.96) 12 months post a cardiovascular event [8]. This result is further supported by another systematic review, which illustrated that patients who attended CR after suffering from myocardial infarction had a lower overall risk of mortality, compared to those who had a lower attendance (OR 0.74; CI 0.58–0.95) [9]. Attending CR is important, as this programme can help mitigate the burden of CVD globally through secondary prevention strategies, thus enhancing pathological and psychological health benefits in patients [5].

Pharmacists play a crucial role in the management and further prevention of CVD by educating patients on the importance of medications, counselling on drug-safety management, encouraging adherence, conducting medication reviews and optimisation, whilst controlling cardiovascular risk factors, which are essential to effective CVD management [10–12]. A non-randomised intervention study conducted in 2004 showed that for every dollar spent on services delivered by pharmacists in CR, there was a potential cost saving of $13.50 [12]. Despite the significant improvement in health outcomes of patients in various other diseases including heart failure through the provision of clinical pharmacist-led interventions, the evidence of pharmacists’ contributions in CR is still limited [12]. Although there has also been a literature review pertaining to pharmacist’s role in CR such as conducting medication reviews, educating patients, and optimising drug therapy, which was completed in 2005 [13], a systematic review specifically exploring the impact of pharmacist interventions in CR on patient outcomes is still needed.

### Aim

This review aimed to explore the role of pharmacists in CR, the impact of their interventions on clinical and non-clinical patient outcomes, and prospects of future role development.

## Method

A protocol was registered with PROSPERO International prospective register of systematic reviews (registration number: CRD42021291716). The systematic review was conducted following the PRISMA guidelines and statement [14].

### Search strategy and inclusion criteria

A systematic search was performed on six databases—Embase, Medline, Cochrane Library, PubMed Central UK, Cumulative Index to Nursing and Allied Health Literature (CINAHL), and PsycINFO for relevant literature published in the period of January 2006 until October 2021 (as a previous review pertaining to pharmacist’s role in CR was completed in 2005). The search used two main keywords, ‘pharmacist’ and ‘cardiac rehabilitation’ with restrictions applied to English language and randomised controlled trials (RCTs), non-randomised controlled trials, and interventional studies with other designs. MeSH terms were used to generate additional keywords to expand our search and identify additional studies for inclusion. The full search terms and key words used are shown in the supplementary material. Studies were deemed suitable for inclusion if they involved adult participants (18 years of age or above) and assessed the role of pharmacist in CR on both clinical and non-clinical patient outcomes. Reference lists of included papers were hand searched and any duplicates were removed.

### Study selection and data extraction process

Titles and abstracts of the records from the search were screened to exclude irrelevant studies, and full texts of the remaining records were retrieved, and reviewed against inclusion and exclusion criteria. The identification and selection process of relevant studies was conducted by AA and ZJ independently, and then the outcomes were discussed. Any discrepancies were resolved through discussion with PG. For each study that was included in this review, information was extracted on year of publication, study design, country of origin, sample size, characterisation of participants, pharmacist-led interventions used and the impact on outcomes.

### Outcomes assessed

The primary outcomes assessed were the role and types of interventions delivered by pharmacists during the CR programme. Secondary outcome measures included the impact of pharmacist-led interventions on patient outcomes including both clinical (e.g. systolic and diastolic blood pressure (SBP/DBP), low-density lipoprotein cholesterol (LDL-C), fasting blood glucose (FBG) and non-clinical outcomes (e.g., medication adherence, HRQoL, and knowledge).

### Quality assessment

The Cochrane risk-of-bias tool was used to assess the quality of RCTs [15]. Each domain of the Cochrane risk-of-bias tool was determined as either having a low, unclear or high risk of bias. The risk of bias summary for RCTs was generated using RevMan software version 5.4 [16]. For the other studies with non-RCT design, the Joanna Briggs Institute (JBI) Critical Appraisal Tool for Quasi-Experimental Studies and the National Heart, Lung and Blood Institute (NIH) quality assessment tool were used to assess quality [17, 18].

## Results

**Fig. 1:**
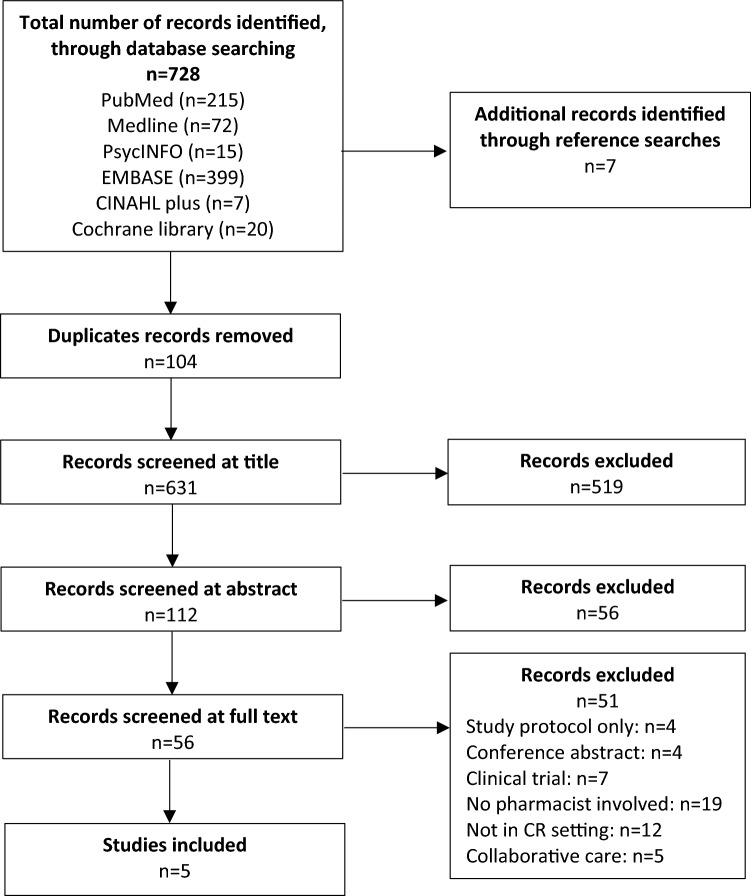
Adapted PRISMA flow chart describing the study selection process

A total of 728 records were accumulated from the electronic search of databases and further seven records were identified through the handsearching of references. After removing duplicates and screening titles and abstracts, the full text of 56 papers were reviewed, of which only five studies met the inclusion criteria and were included in this review (Fig. 1). A meta-analysis was considered; however, it was not possible as there was considerable heterogeneity and variability amongst the studies. Therefore, a narrative synthesis was conducted.

### Study characteristics

Study characteristics are described in Table 1. The studies were conducted in different countries, each from Egypt [19], Malaysia [20], Canada [21], Sweden [22], and United Kingdom [23]. Sample sizes ranged from 40 to 316 patients and follow-up periods ranged from 3 to 15 months [19–23]. Interventions were conducted by pharmacists only, on a twice weekly basis [19], weekly [23], fortnightly [20], at three months and then ten months post discharge [22], and on a weekly basis for two weeks and then when required within a six-month time frame [21].

**Table 1:** Study characteristics for included studies

Study ID	Study design	Length of intervention	Cardiac Rehabilitation (CR) phase	Sample size	Conditions treated
Casper et al. [19] 2017, Egypt	Prospective RCT	3-month follow up duration	CR Phase II	Intervention group: 20	NSTEMI: 35
				Control group: 20 (*n* = 40)	STEMI: 5
Anchah et al. [20] 2008–2010, Malaysia	Pre-post quasi experimental non-equivalent study	12-month follow up duration	CR Phase I and II	Intervention groups:	NSTEMI: 25
				MCRP: 22	STEMI: 61
				CCRP: 28	Unstable angina: 32
				Control group: 62 (*n* = 110)	
Alsabbagh et al. [21] 2009–2010, Canada	Open label RCT	6-month follow up duration	CR Phase II	Intervention group: 46	NSTEMI: 4
				Control group: 48 (*n* = 95)	STEMI: 3
					Stent: 19
					CABG: 20
					NSTEMI + stent: 10
					STEMI + stent: 30
					NSTEMI + CABG: 5
					STEMI + CABG: 3
Ostbring et al. [22] 2013–2016, Sweden	Prospective RCT	15-month follow up duration	CR Phase II	Intervention group: 159	NSTEMI: 91
				Control group: 157 (*n* = 316)	STEMI: 94
					Stent: 19
					Unstable angina: 35
					Chronic angina: 69
					Other: 25
Packard et al. [23] 2008–2010, UK	Non-randomised intervention study	Not mentioned	CR Phase II	(*n* = 192)	Not mentioned

*RCT* Randomised controlled trial, *CR* cardiac ehabilitation, *MCRP* modified cardiac rehabilitation, programme (intervention group), *CCRP* conventional cardiac rehabilitation programme, *STEMI* ST-elevated myocardial infarction, *NSTEMI* non-ST elevated myocardial infarction, CABGcoronary artery bypass graft

### Study quality

**Fig. 2:**
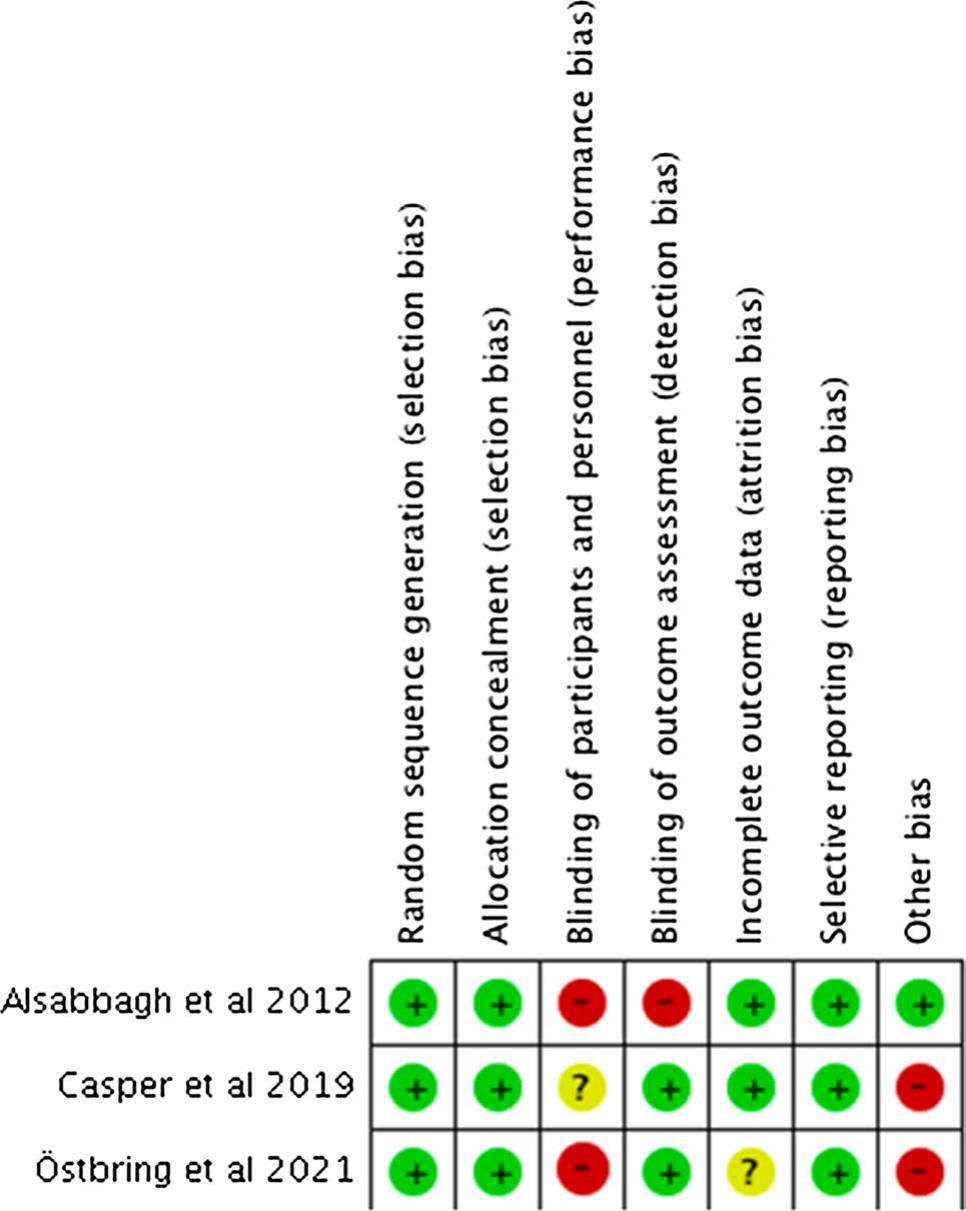
Risk of bias summary graph and the outcome of each domain for the RCT studies

**Fig. 3:**
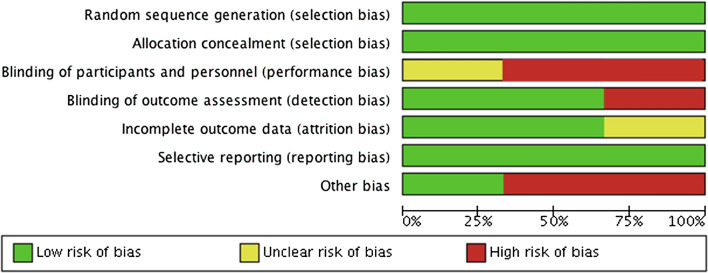
Risk of bias graph of the RCT studies presented as percentages for each risk of domain

Random sequence generation and allocation concealment were adequately reported amongst all three RCTs [19, 21, 22]. However, due to the nature of the intervention, blinding the participants and pharmacists was not possible, hence either a high risk [21, 22] or unclear risk of bias was noted. In addition, a low risk of bias was determined for incomplete outcome data in two studies, as reasons for patient dropouts were justified in the review and missing data was accounted for in the results [19, 21]. In the study conducted by Ostbring et al. [22] a high risk of attrition bias was found because most patients missing were from the intervention group, which could have reduced the validity of results for adherence. Risk of bias for selective reporting was low across all RCTs, as they reported the outcomes they intended to as per their trial protocol. However, the potential risk of other bias was found to be high due to single-centre design [19], follow-up duration [19, 21, 22], small sample size [19], and difference in patient demographics across the intervention and control group [19, 21, 22] (Figs. 2, 3). The study that was assessed using the JBI critical appraisal tool was found to be of good quality [20], and the other study assessed by the NIH tool was found to be of fair quality [23].

### Setting of CR programmes

Pharmacist interventions were directly delivered to patients in outpatient hospital settings in four studies [19–22], and one study conducted pharmacist interventions over the telephone [21]. Out of the five studies included, only one study reported the outcomes of pharmacist interventions in both Phase I (the initial phase of inpatient) and Phase II of CR programme (the continuation phase of outpatient) [20], whilst the other four studies [19, 21–23] only assessed the impact in phase II. Phase I CR programme is an inpatient programme delivered to patients during their hospital stay following a cardiac event or procedure, and phase II is delivered to patient’s post-discharge from hospital [20].

### Pharmacist interventions

Interventions were provided by pharmacists in four studies [19–22], and in one study, it was delivered by undergraduate pharmacy students under supervision [23]. Pharmacists provided a variety of different interventions including: patient education, medication review and optimisation, identification and resolution of drug-related problems (DRPs), encouragement of medication adherence, and lifestyle modifications [19–23] (Table 2).

**Table 2:** Studies evaluating pharmacist’s intervention in cardiac rehabilitation on patient outcomes

Study ID	Pharmacist interventions	Outcome Measures	Results
Casper et al. [19] 2017, Egypt	Patient education	• Medication adherence (MMAS-8)	A significant improvement between the intervention and control. Number of patients achieving target LDL-C levels in the intervention group were found to be statistically significant (*P* = 0.0084)
	Medication adherence	• Patients knowledge (CADE-Q)	
	Identification and resolution of DRPs	• HRQoL (SF-36)	
		• Drug related problems (DRP)	
		• HR, SBP, DBP, LDL, TC and FBG	
Anchah et al. [20] 2008–2010, Malaysia	Intensive drug counselling	• Pre-post health related quality of life (SF-36 questionnaire)	At the 12-month follow up, an improvement in the physical and social functioning (SF) component of HRQoL questionnaire was found in the intervention group, compared to control group (mean difference: 11.46 (*P* = 0.008), 10.96 (*P* = 0.002) and 6.41 (*P* = 0.006)) and (mean difference in SF: 20.53, 14.47 and 8.8 (*P* = 0.03)
	Medication adherence		
	Lifestyle and diet modification		
	CVD risk factor reduction		
Alsabbagh et al. [21] 2009–2010, Canada	Telephone-based CR	• Mean medication adherence (MPR—medication possession ratio)	There was no difference in medication adherence, between the control and intervention group (88.8% vs 89.9%)
	Patient education and counselling		
	Medication adherence		
	Barriers to medication adherence		
Ostbring et al. [22] 2013–2016, Sweden	Medication review Motivational interviewing – used to identify barriers to medical adherence	• Target LDL-C (< 1.8 mmol/L) by 12 months	More patients in the control group reached the target goal for LDL-C (37 and 44% respectively)
		• Medication adherence (MMAS-8)	
		• Systolic blood pressure	
	Drug related problems identification and resolution	• Disease QoL	
Packard et al. [23] 2008–2010, UK	Medication reconciliation	• Cardiac rehabilitation pharmacy services form	Most common interventions included: changing administration time of medication (36.8%) and avoidance of significant drug interactions (33.3%)
	Patient counselling		
	Medication adherence	• Pharmacy communications form	

*CADE-Q* Coronary artery disease education questionnaire, *QoL* quality of life, *HRQoL* health related quality of life, *MMAS-8* Morisky medication adherence scale, *SBP* systolic blood pressure, *DBP* diastolic blood pressure, *FBG* fasting blood glucose, *HR* heart rate, *TC* total cholesterol, or *LDL-C* low-density lipoprotein cholesterol levels, *CR* cardiac rehabilitation, *MCRP* modified cardiac rehabilitation, programme, *CCRP* conventional cardiac rehabilitation programme

### Outcomes

Four studies reported the impact of pharmacist interventions on at least one patient outcome [19–22]. Two studies reported the number of drug-related problems identified by pharmacists [19, 23]. Outcomes of each study are summarised in Table 3.

**Table 3:** The effect of pharmacist interventions in addition to CR on clinical outcomes

Study ID	Clinical outcome measured + units	Effect of Pharmacist interventions in CR on clinical outcomes of patients	*P*-Value
Casper et al. [19] 2017, Egypt	HR (bpm)	A statistically significant reduction in HR was observed in intervention group compared to the control group, from baseline to after 3 months (mean percentage change ± SD: (− 10.04 ± 8.406) and (6.791 ± 10.88) *P* = 0.001) respectively. However, the number of patients achieving the target HR goal of 60–100 bpm in the intervention and control group (35 and 20%) was statistically insignificant	0.4801
	BP (mmHg)	A statistically significant decrease in both SBP and DBP was observed in the intervention group compared to the control group, from baseline to after three months: (mean percentage change in SBP ± SD (− 16.22 ± 9.987) vs (4.751 ± 15.52) *P* = 0.0001) and (mean percentage change in DBP ± SD (17.87 ± 15.09) vs (10.45 ± 18.57) *P* = 0.0001). However, the number of patients achieving the target BP goal of < 130 mmHg, in the intervention ad control group (85 and 65%) was statistically insignificant	0.2733
	LDL-C (mg/dL)	A statistically significant decrease in LDL-C was observed in the both the intervention and control group, from baseline to after three months [median percentage change ± IQR: − 25.7 (− 38.8 to − 7.7) vs − 0.253 (− 24.2 to 41.7) *P* = 0.0071] respectively. Also, the number of patients achieving the target goal of LDL-C < 70 mg/dL, in the intervention group compared to the control group (80 and 60% respectively) was statistically significant	0.0084
	TC (mg/dL)	A statistically significant decrease in TC was observed in the both the intervention and control group from baseline to after three months [median percentage change ± IQR: − 14.6 (− 26.6 to − 2.2) vs 4.123 (− 9.4 to 12.06) *P* = 0.0005]. However, the difference between the number of patients achieving the target TC goal of < 200 mg/dL, in the intervention group compared to the control group (100 and 90%) was found to be statistically insignificant	0.4872
	FBG (mg/dL)	A statistically significant decrease in LDL-C was observed in the both the intervention and control group from baseline to after three months [median percentage change ± IQR: 11.4 (− 38.6 to − 0.2) vs 5.4 (− 8.21 to 14.06) *P* = 0.0098]. However, the difference between the number of patients achieving the target FBG goal of 80-130 mg/dL, in the intervention group compared to the control group (85 and 80%) was statistically insignificant	1.0000
Ostbring et al., [22] 2013–2016, Sweden	LDL-C (mmol/L)	No statistically significant difference was observed for LDL-C levels at 12 months post discharge in the intervention group compared to the control group. The proportion of patients that reached the target LDL-C goal of < 70 mg/dL (1.8 mmol/L), by 12 months after discharge, was greater in the control group than in the intervention group (37%, vs 44.2% respectively) with a 95% CI (− 7.2 (− 19.9–5.3)). However, it was observed that more patients that were adherent to their cholesterol lowering medication achieved the target goal LDL-C, compared to those who were non-adherent	0.2630
	SBP (mmHg)	The proportion of patients who achieved the target SBP goal of < 140 mmHg in the intervention group compared to the control group was found to be statistically insignificant. In the intervention group 59.5% of patients achieved the target goal for SBP, whilst in the control group only 58.3% of the patients met the target goal [95% CI = 1.1 (− 11.9 to 14.2)]	0.8650

*HR* Heart rate, *bpm* beats per minute, *BP* blood pressure, *SBP* systolic blood pressure, *DBP* diastolic blood pressure, *LDL-C* low-density lipoprotein cholesterol, *TC* total cholesterol, *FBG* fasting blood glucose, *IQR* interquartile range

#### Medication adherence

Pharmacist provided services were evaluated in three studies [19, 21, 22], however, methods of measuring adherence varied. Self-reported adherence was assessed using Morisky Medication Adherence Scales (MMAS-8) [19, 22], and mean medication adherence was determined through Medication Possession Ratio (MPR) [21]. Prescription refill adherence was used to classify patients according to their medication compliance behaviours and calculate the proportion of days covered (PDC) [22]. Only two studies demonstrated a statistically significant improvement in medication adherence, following pharmacist-led interventions in addition to CR [19, 22].

#### Health related quality of life (HRQoL)

Three studies analysed the impact of pharmacist interventions on patients HRQoL, following a cardiovascular event, procedure or CVD diagnosis [19, 20, 22]. The generic quality of life assessment tool: SF-36 was used to determine the burden of CVD on patient’s health in two studies [19, 20] and the disease-specific tool HeartQoL was used in one study [22]. In the studies conducted by Casper and Anchach et al., a significant increase in the physical health component was observed in the intervention group, from baseline to after 3 months (median score 37.5 (0–75) to 100 (75–100)) [19] and from baseline to after 12 months (mean difference 11.46 (*P* = 0.008) and 6.41 (*P* = 0.006) in the control group) respectively [20].

#### DRP identification and resolution

The incidence of DRPs was reported in two studies [19, 23]. A total of 138 drug-related interventions were made by pharmacists in the study conducted by Casper et al. with an acceptance rate of 96.2%. Similarly, in the study conducted by Packard et al., 467 drug-related interventions were made, of which 79.9% of those interventions did not require a physician’s response. Patient interventions recommended by pharmacists included the management of drug side effects, drug-addition, dose adjustment [19], and avoidance of significant drug interactions [23].

#### Clinical outcomes

LDL-C levels and BP was assessed in two studies [19, 22], however only one study reported a statistically significant (*P* < 0.05) improvement in LDL-C levels, following pharmacist intervention [19].

## Discussion

Despite advances in medical care, CVD remains a significant global healthcare problem and its management includes a variety of different aspects of care. A multidisciplinary approach is therefore crucial for its treatment [24]. The results of this review need to be approached with caution due to the small number of studies found, however, the value of pharmacist interventions in the management of patients with CVD was demonstrated, through a statistically significant improvement in patient’s medication adherence [19, 21, 22], HRQoL [19, 20, 22], patient’s knowledge on CVD [19], and a reduction in LDL-C levels to the optimal target range of < 1.8 mmol/L [19]. The most frequently used interventions that were provided by pharmacists exclusively included patient education sessions [19–21, 23], review of patients’ knowledge on both CVD and medication [19–21], DRP identification and resolution [19, 23], medication reconciliation and review [17–21], lifestyle modifications [17], and encouragement of medication adherence [19–23]. The significance of pharmacist interventions is concurrent with several other studies.

A study conducted by Sharma et al., illustrated that patients who received services from allied health practitioners including pharmacists, had significantly lower Total Cholesterol (TC), LDL-C and triglyceride levels at 12 months, and greater medication adherence in the intervention group compared to the control group up to 24-months post-intervention [25]. Furthermore, a review by Davis et al., demonstrated that pharmacist interventions are effective and have long-lasting beneficial and protective effects on patients with CVD [26]. These findings are also supported by a systematic review of 12 RCTs where pharmacist care led to greater adherence to angiotensin converting enzyme inhibitors and beta-blockers, and was also found to be associated with a reduction in all-cause hospitalisations (OR 0.71, 95% CI 0.54–0.94, *P* = 0.02) [27].

The complexity and burden of medical therapy in patients with CVD, misconceptions and patient’s attitudes to medication are profound factors of non-adherence [28, 29]. Non-adherence is a preventable cause of mortality in patients with CVD, however, remains a significant hurdle in improving patient outcomes. Several studies have demonstrated that disease specific risk of mortality increases with non-adherence, therefore strategies to address this are crucial [27, 30, 31]. Pharmacist interventions, in addition to standard CR, demonstrated a favourable change in patient’s adherence to treatment, through the provision of tailored patient education along with repeated reinforcement, either via direct patient contact [19] or over the telephone [21]. This improvement in medication adherence is synonymous with other studies, they found that better medication adherence and disease control was observed following pharmacist-led intervention compared to patients receiving standard care [32–34]. A few studies have also demonstrated that motivational interviewing techniques lead to greater improvement in patients medication adherence, HRQoL, and a reduction in LDL-C levels [35, 36]. Therefore, the most effective pharmacist interventions that promote behaviour change are found to be multifaceted [37], targeted and personalised to the patient’s needs and beliefs [38, 39]. These results support the positive impact of Pharmacist care in CR and the use of motivational interviewing as a fundamental technique to optimise patient’s adherence to medication [40] and improve their HRQoL [41–45].

It is notable that management of CVD is complex, therefore pharmacist interventions and services should be established and standardised in CR settings to ensure treatment efficacy, safety, and medication adherence.

### Strengths and limitations

Studies that assessed the impact of pharmacists alone, not as part of collaborative care, were included and the review was not limited to any outcome measures. This ensured specificity and made it easier to delineate the outcomes and relate it to the role of pharmacists in CR. Nevertheless, this review has some limitations, although the search was very specific, this review did not search for grey literature and studies published in languages other than English. Additionally, there was a considerable clinical and statistical heterogeneity between the identified studies; the duration and follow-up period varied between the studies, as did the outcomes assessed; therefore, limited our ability to draw any robust conclusions on the effectiveness of pharmacist interventions. Self-reported adherence questionnaires were used as a measure of medication adherence, although they are an acceptable and valid measure of adherence, they can overestimate the level of medication adherence, if patients answer the questions untruthfully. Furthermore, as most of the studies had either an unclear or high risk of bias, the possibility of publication and selection bias should be taken into consideration and thus the positive results from the study should be interpreted carefully.

### Future research

This review demonstrated encouraging and beneficial evidence for the further integration of face-to-face multi-factorial weekly pharmacist provided services, with frequent follow-ups, in addition to the standard CR programme. Given the increasing complexity of CVD management, pharmacists’ unique focus on patient education, medication review and reconciliation should be considered when managing CVD and hence continuity of pharmacist provided services is important [46, 47]. However, future research is needed to explore the effectiveness of pharmacist interventions on other medical and non-medical risk factors of CVD, such as diabetes and smoking, and the feasibility of pharmacist provided services. Economic evaluations are necessary to prove the cost-effectiveness of pharmacist provided services to healthcare systems, patients, and society including hospitalisation and mortality rates.

## Conclusion

The evidence from this study is promising and demonstrates that pharmacist interventions in addition to the standard CR programme can play an important role in the secondary prevention of CVD, by improving patients health outcomes and quality of life, managing and preventing DRPs and promoting lifestyle changes.

The expanding scope of pharmacist’s role has favourable effects on patients, therefore these findings support the need for greater pharmacist involvement in CR. However, due to limited evidence, larger clinical trials in CR settings are required to evaluate the long-term impact of pharmacist interventions on patients post a cardiac event or procedure.

## Supplementary Information

Below is the link to the electronic supplementary material.

Supplementary file1 (DOCX 22 KB)
