# Supplementary material for: A systematic review investigating the role and impact of pharmacist interventions in cardiac rehabilitation
Source: Int J Clin Pharm. 2022 Nov 19;45(2):320–9. doi: 10.1007/s11096-022-01517-1 (PMC10147760; doi:10.1007/s11096-022-01517-1)
Supplement: Supplementary file 1 — Supplementary file1 (DOCX 22 KB) [file 11096_2022_1517_MOESM1_ESM.docx]

| **SEARCH STRATEGY**  A database search conducted in Ovid MEDLINE (1967 to October 2021)  Results: n= 52  Limits: English language and years (2006 to 2021), n=36.   \| [#](https://ovidsp.dc1.ovid.com/ovid-a/ovidweb.cgi?&S=OCDCFPMCFNACBDMAKPOJOHKIDHHDAA00&Sort+Sets=descending) \| Search \| \| --- \| --- \| \| 1 \| “Pharmacy” OR Pharmacist* \| \| 2 \| (“Pharma* intervention”) \| \| 3 \| (Intervention$ adj3 pharma*$) \| \| 4 \| Pharma* service \| \| 5 \| 1 OR 2 OR 3 OR 4 \| \| 6 \| exp Cardiac rehabilitation/ \| \| 7 \| (Cardiac adj3 rehab*) \| \| 8 \| Cardiovascular rehab* \| \| 9 \| “Cardiovascular secondary prevention” \| \| 10 \| heart rehab* \| \| 11 \| 6 OR 7 OR 8 OR 9 OR 10 \| \| 12 \| 5 AND 11 \| |
| --- | --- | --- | --- | --- | --- | --- | --- | --- | --- | --- | --- | --- | --- | --- | --- | --- | --- | --- | --- | --- | --- | --- | --- | --- | --- | --- |
